# Links Between Early-Life Contextual Factors and Later-Life Cognition and the Role of Educational Attainment

**DOI:** 10.1093/geroni/igab046.1214

**Published:** 2021-12-17

**Authors:** Jordan Palms, Laura Zahodne

**Affiliations:** University of Michigan, Ann Arbor, Michigan, United States

## Abstract

Educational attainment is a well-documented predictor of later-life cognition, but less is known about upstream contextual factors. This study aimed to identify which early-life contextual factors uniquely predict later-life global cognition and whether educational attainment mediates these relationships. Participants were drawn from the Michigan Cognitive Aging Project (N=461; Mage=63.51; SDage=3.13; 50% non-Hispanic Black). School-level contextual factors included U.S. region during elementary school (Midwest, South, Northeast), racial diversity of school (mostly White, mostly Black, diverse), self-reported education quality, and school type (public versus private). Household-level contextual factors included mother’s and/or father’s education, number of adults (1, 2, 3+), and number of children. Later-life global cognition was operationalized with a composite score derived from a comprehensive neuropsychological battery. A mediation model controlling for sociodemographics estimated total, direct, and indirect effects of contextual factors through educational attainment (years). Lower education quality, attending a mostly Black or diverse school, attending a public school, and reporting three or more adults in the household were each associated with lower cognition. After accounting for educational attainment, associations remained for education quality, school type, and reporting three or more adults in the household. Indirect effects through educational attainment were observed for elementary school region, education quality, racial diversity of school, and mother’s education. School context appears to more consistently predict later-life cognition than household context, highlighting the potential long-term benefits of school-level interventions for cognitive aging. Future research should consider causal relationships among household-level and school-level contextual factors, as well as additional mediators beyond educational attainment.

